# Proteomic variation and diversity in clinical *Streptococcus pneumoniae* isolates from invasive and non-invasive sites

**DOI:** 10.1371/journal.pone.0179075

**Published:** 2017-06-02

**Authors:** Mustapha Bittaye, Phil Cash, Ken Forbes

**Affiliations:** School of Medicine, Medical Sciences & Nutrition, University of Aberdeen, Aberdeen, United Kingdom; Instituto Butantan, BRAZIL

## Abstract

*Streptococcus pneumoniae* is responsible for a variety of invasive and non-invasive human infections. There are over 90 serotypes of *S*. *pneumoniae* differing in their ability to adapt to the different niches within the host. Two-dimensional gel electrophoresis was used to discriminate clinical *S*. *pneumoniae* isolates recovered from either blood cultures (invasive site isolates) or other sites, including sputum, tracheal aspirate, ear, eye and skin swabs (non-invasive site isolates). Global protein expression profiles for five invasive site and six non-invasive site isolates representing five different serotypes (serotypes 4, 6, 9, 14 and 23) were obtained for each isolate and combined into a single data set using Progenesis SameSpots^™^ software. One-hundred and eighty six protein spots (39% of the protein spots in the dataset) differed significantly (ANOVA, p<0.05) in abundance between the invasive site (101 upregulated protein spots) and non-invasive site (85 upregulated protein spots) isolates. Correlations between the bacterial proteomes and their sites of isolation were determined by Principal Component Analysis (PCA) using the significantly different protein spots. Out of the 186 variable protein spots, 105 exhibited a serotype-associated pattern of variability. The expression of the remaining 81 protein spots was concluded to be uniquely linked to the site of bacterial isolation. Mass spectrometry was used to identify selected protein spots that showed either constant or differential abundance levels. The identified proteins had a diverse range of functions including, capsule biogenesis, DNA repair, protein deglycation, translation, stress response and virulence as well as amino acid, carbohydrate, lipid and nucleotide metabolism. These findings provide insight on the proteins that contribute towards the adaptation of the bacteria to different sites within the host.

## Introduction

*Streptococcus pneumoniae* (also known as the pneumococcus) is a significant human pathogenic bacterium that remains a leading cause of morbidity and mortality worldwide. The pneumococcus is the principal bacterial cause of community-acquired pneumonia and acute otitis media as well as being associated with several other non-invasive infections including sinusitis and conjunctivitis [1]. The human nasopharynx is the natural ecological niche of the bacterium, where it exists asymptomatically in a carrier state. Migration of the bacterium across the respiratory epithelium into the blood system and eventually the cerebro-spinal fluid (CSF) leads to the development of severe life-threatening, invasive pneumococcal diseases (IPDs) such as sepsis and meningitis [2]. Worldwide, pneumococcal infections are responsible for the deaths of 1.6 million people annually [3]. Young children, particularly in low-income countries, the elderly, the economically deprived as well as those with HIV/AIDS bear a disproportionate share of the disease burden. The highest mortality due to pneumococcal infection occurs in patients who develop pneumococcal sepsis and meningitis [4]. Immunization and antimicrobial therapy are the cornerstones for the prevention and management of pneumococcal infections. However, the lack of a universal vaccine against the many circulating pneumococcal serotypes, as well as the widespread prevalence of antibiotic resistance, present specific challenges for disease control and management [5]. These obstacles are compounded further by a lack of rapid, sensitive, and specific diagnostic tests for IPD.

*S*. *pneumoniae* is a genetically diverse species that exists in many different capsular and sequence types. The ability of the bacterium to cause either invasive disease or localised infections varies markedly between individual isolates. This pathogenic variability is influenced by both the bacterial capsular serotype and genetic background [6,7]. Moreover, the bacterial site of isolation has also been shown to influence the virulence behaviour displayed by the bacterium [8,9]. Comparative studies on the available genome sequences of *S*. *pneumoniae* isolates identified core genes that are common to all isolates as well as accessory regions comprising variable genes that correlate with virulence phenotype. Both the core genome and regions of diversity encode virulence determinants whose expression varies according to the bacterial serotype and sites of infection [6,7]. This dynamic nature of the pneumococcal expression of virulence factors in different host niches has been investigated in recent years using comparative transcriptomics. Both Ogunniyi *et al*. [8] and Orihuela *et al*. [9] reported niche-specific differences in the transcriptional profiles of pneumococci recovered from the nasopharynx, lung and blood following intranasal infection of mice. However, these transcript profiling studies have been carried out on commonly used laboratory-adapted *S*. *pneumoniae* strains, in mice, and cannot consider the expression levels of the related proteins and their post-translational modifications.

The comparison of pathogenic microorganisms at the proteomic level provides complementary data to those obtained using nucleic acid based methods. The availability of complete genome sequences for many virulent and avirulent strains of *S*. *pneumoniae* provides the basis for in depth investigations of the pneumococcal proteome, particularly for studies on pneumococcal pathogenesis [5]. To date, several proteomic studies on *S*. *pneumoniae* have been reported that focus primarily on identifying potential virulence determinants expressed by laboratory type strains of pneumococcus grown *in vitro* under conditions that mimic specific host environments [10–13]. However, less attention has been paid towards comparative proteomic analyses of clinical *S*. *pneumoniae* isolates recovered from different host niches in order to identify markers correlating with specific-disease phenotypes as well as obtaining information on pathogenic differences arising from strain variation.

In the following study, a global comparative proteomics approach using two-dimensional gel electrophoresis (2-DE) based protein profiling was employed to differentiate invasive and non-invasive site clinical *S*. *pneumoniae* isolates. The combination of 2-DE coupled with mass spectrometry (MS) for protein separation and identification respectively, have been extensively employed to study the pathogenesis for a number of important human bacterial pathogens [14–17]. In this study, we compared invasive site pneumococcal isolates recovered from blood cultures with non-invasive site isolates recovered from various mucosal surfaces (sputum, tracheal aspirate, ear, eye and skin swabs), in order to investigate the proteome variation and diversity associated with the pneumococcal isolates from the two infection sites. Despite the extensive heterogeneity found for the pneumococcal isolates, it was possible to discriminate the invasive and non-invasive site isolates on the basis of their 2-dimensional (2-D) protein profiles. Moreover, the proteins that displayed relatively constant abundance levels across the pneumococcal isolates could in the long run be investigated for inclusion in the pipeline of potential vaccine markers. Thus, the pan proteomic analysis of multiple pneumococcal isolates recovered from invasive and non-invasive sites of infection is a valuable approach for identifying proteins involved in pneumococcal colonisation and virulence.

## Results

### Growth of *S*. *pneumoniae* in BHI-FCS broth

**Fig 1:**
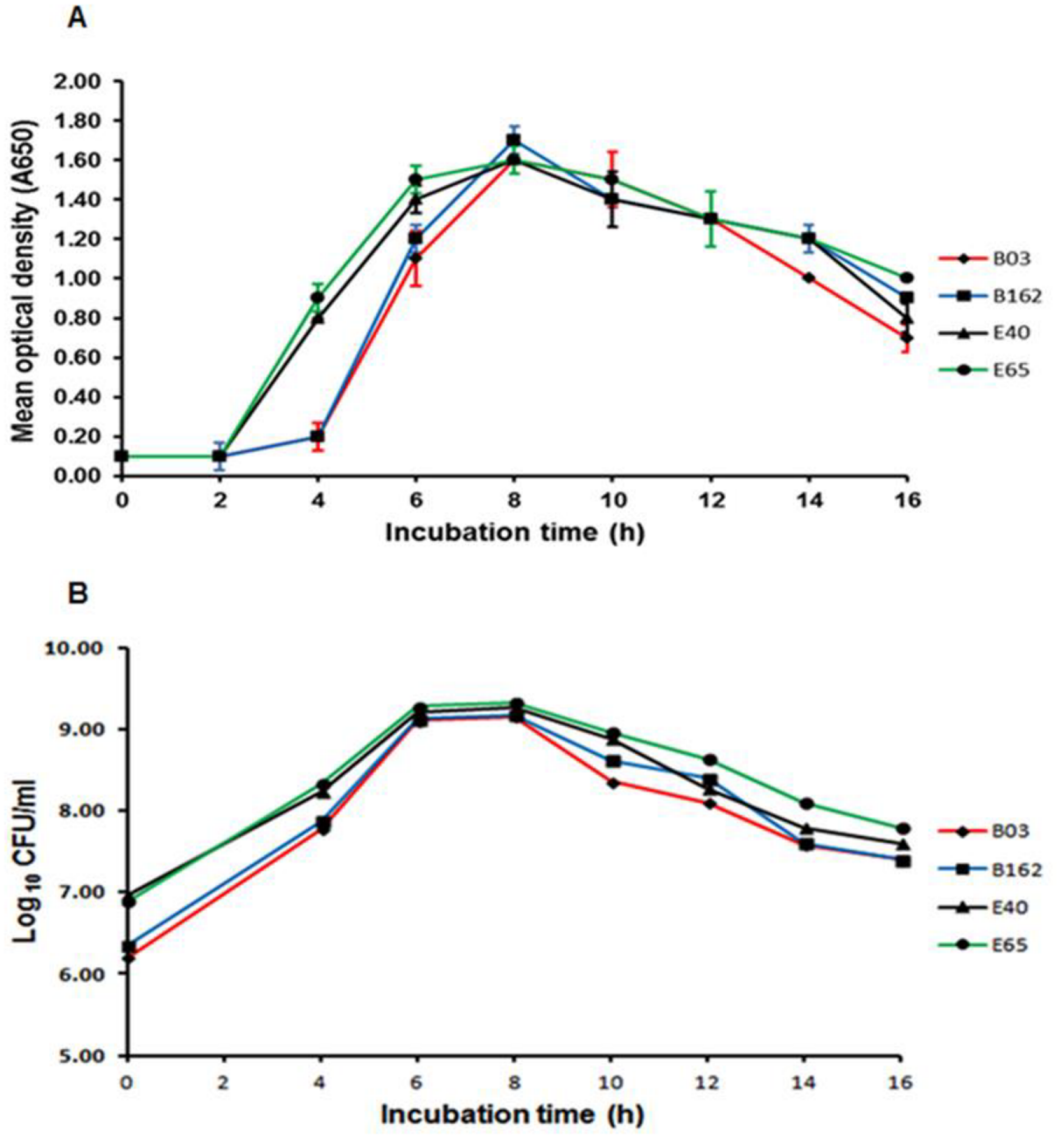
Characteristic growth and viability curves of representative *S*. *pneumoniae* isolates in BHI-FCS broth. **(A)** Measurement of the bacterial cell density **(B)** Measurement of bacterial viability.

To optimise the bacterial culture conditions for the analysis of the pneumococcal cellular proteins, representative isolates were grown in BHI-FCS broth and assayed for bacterial cell density and viability as described in the Materials and methods section. The growth curves for two invasive site isolates (B03 and B162) and two non-invasive site isolates (E40 and E65) are shown in Fig 1. Under the experimental conditions used, the four pneumococcal isolates showed similar growth characteristics with the lag-phase for each isolate lasting at least 2 h (Fig 1A), which was followed by an increase in the bacterial cell density. All of the isolates reached their maximum cell density at 8 h post-inoculation followed by a decline in cell density thereafter. The cell viability followed a similar pattern as cell density, although no clear lag phase was identifiable (Fig 1B). Based on these data, the bacterial cells were routinely collected once the inoculated cultures had reached an OD650 of 1.0 to ensure that sufficient biomass (approximately 3 x 10^7^ CFU/mL) was obtained for subsequent proteomic analysis.

### Comparison of the pneumococcal 2-D protein profiles

To identify those pneumococcal proteins that differed significantly in abundance levels between the invasive and non-invasive site isolates, the isolates were classified into two groups according to their site of isolation (“invasive site” or “non-invasive site”) (Table 1). The cellular proteins for each of the pneumococcal isolates shown in Table 1 were prepared and analysed for 3 biological replicates; representative 2-D protein profiles for each isolate are presented in S1 Fig. Following a manual review of the spot detection data, a total of 483 protein spots was detected for the 33 matched 2-D protein profiles comprising triplicate 2-D gels for each of the 11 clinical pneumococcal isolates. The majority of the detected protein spots had isoelectric points (*pI*) between 4.5 and 6 and molecular weights (*Mr*) range between 20 kDa to 55 kDa. Principal Component Analysis (PCA) was used to determine the degree of variation between the pneumococcal isolates and to identify those proteins that discriminated the two groups.

**Table 1:** Clinical *S*. *pneumoniae* isolates used in the study.

Isolate Category	Isolate ID	Serotype	Site of Isolation
**Invasive**	B03	14	Blood culture
	B40	4	Blood culture
	B154	6	Blood culture
	B162	9	Blood culture
	B269	4	Blood culture
**Non-invasive**	E22	23	Eye swab
	E35	14	Sputum
	E40	14	Skin swab
	E47	23	Sputum
	E65	9	Ear swab
	E107	6	Tracheal aspirate

**Fig 2:**
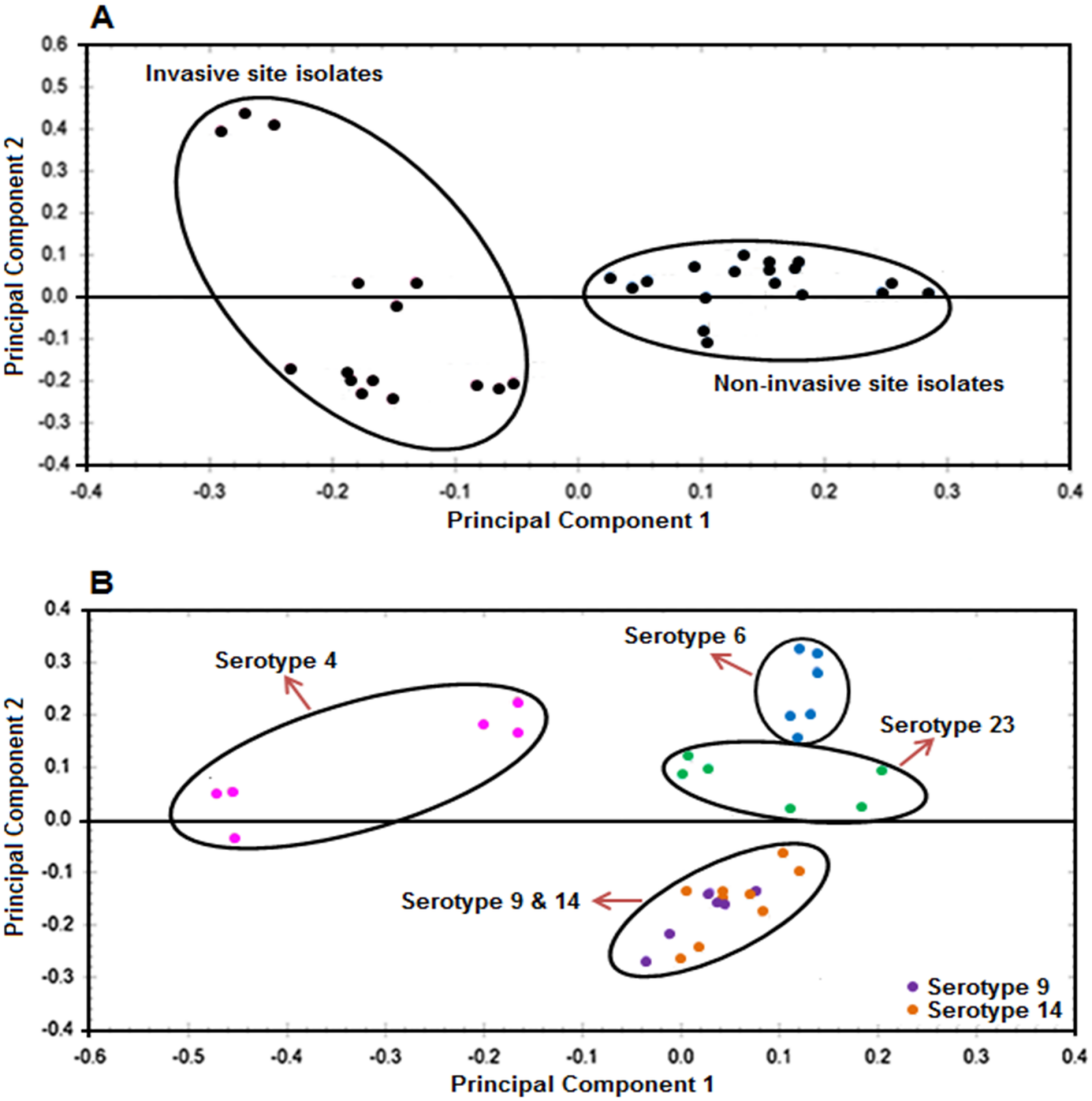
Principal component analysis of the 2-D gel data. The PCA graphs displayed the clustering of the bacterial isolates grouped according to their **(A)** sites of isolation and **(B)** capsular serotype. The PCA was plotted using those protein spots that differed significantly in abundance levels (ANOVA, p<0.05) between isolates in the classified groups.

PCA failed to differentiate the invasive and non-invasive site pneumococcal isolates when all of the 483 detected protein spots were used for the analysis (data not shown). Based on the study of Smith *et al*. [15] PCA using those protein spots (186 protein spots) which differed significantly (ANOVA, p<0.05) in abundance between the two groups of pneumococcal isolates clearly discriminated the two groups of isolates (Fig 2A). The PCA showed that the expression profiles of 101 and 85 protein spots defined the invasive and non-invasive *S*. *pneumoniae* isolate groups respectively. These discriminatory protein spots (101 and 85 in the invasive and non-invasive groups respectively) showed high abundance levels in the respective groups. Based on the clustering of the 2-D protein profiles, those pneumococcal isolates within the invasive site group appeared to be more heterogeneous at the proteomic level than those isolates within the non-invasive site group (Fig 2A).

The eleven pneumococcal isolates analysed in this study represented five different serotypes (Table 1). Since capsular serotype plays a key role in pneumococcal pathogenesis [18], it was possible that the capsular serotypes might contribute towards the discrete groups identified above by PCA (Fig 2A). To test this, the 2-D gel images were grouped according to the pneumococcal serotypes, producing five groups. Two hundred and sixty six protein spots were identified that showed significant differences (ANOVA, p<0.05) in abundance between the five groups and PCA discriminated the isolates on the basis of serotype (Fig 2B). The pneumococcal isolates of serotypes 4, 6 and 23 formed separate, unique clusters, whereas serotypes 9 and 14 formed a fourth cluster within the analysis (Fig 2B). The pneumococcal isolates collected from the two general host niches indicated in Table 1 were distributed across each of the four clusters identified in Fig 2B.

**Fig 3:**
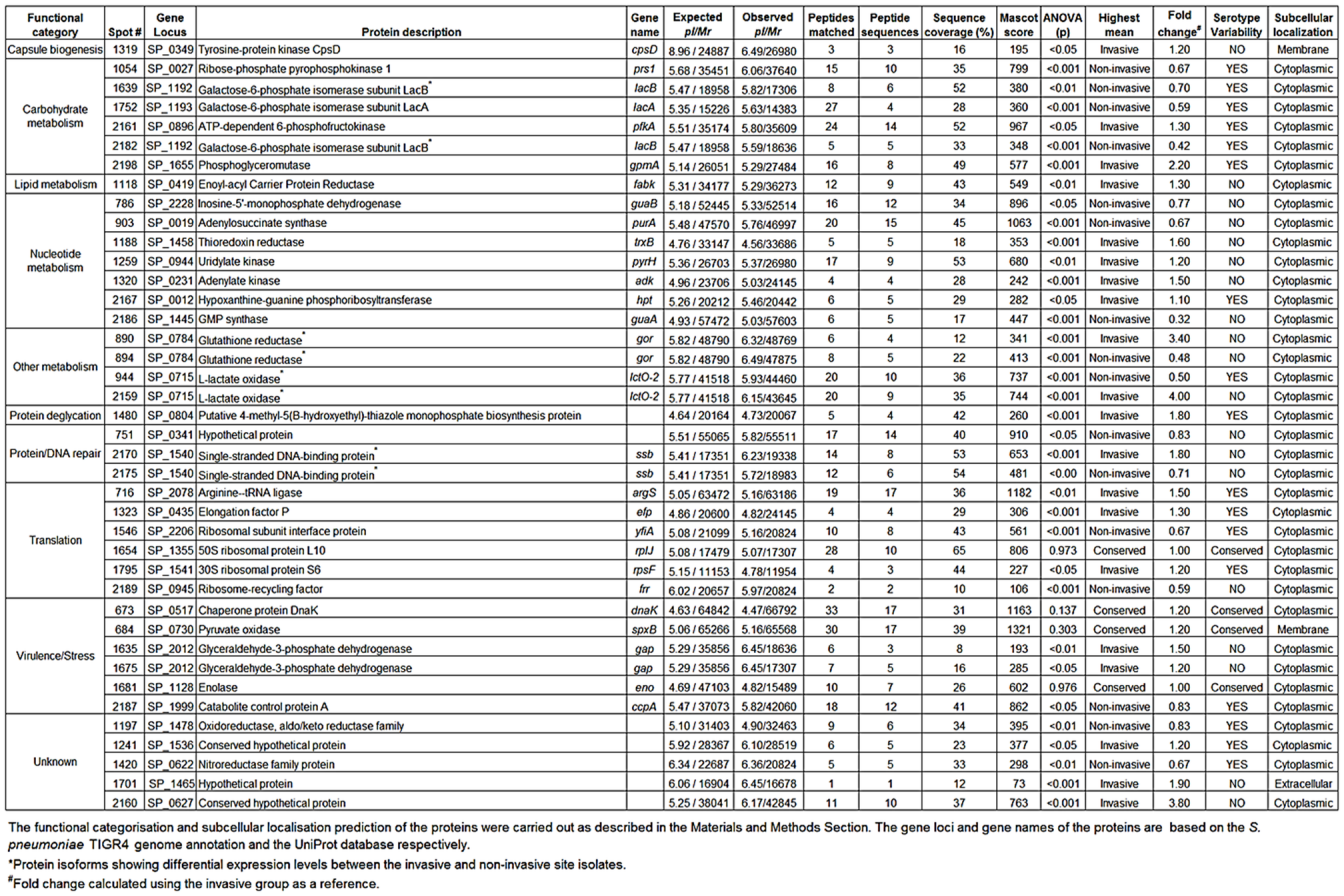
*S*. *pneumoniae* proteins showing constant and differential expression between the invasive and non-invasive site isolates.

Within the complete dataset of 483 protein spots, a greater proportion of the protein spots differed in abundance among the serotypes (266, 55%) than between the sites of isolation (186, 38.5%). Eighty-one of the proteins that exhibited significant differences between the sites of isolation were not significantly different among the serotypes. These 81 proteins spots, which uniquely discriminated the pneumococcal isolates by site of isolation (i.e. that conferred no serotype variability), were of particular interest since they might play a role in the colonisation and invasion of specific niches within the host during infection. Nineteen of these protein spots were identified by LC-MS (Fig 3); 12 of the protein spots showed increased abundances in the invasive site isolates and 7 protein spots showed the highest abundance in the non-invasive site isolates (Fig 3).

### Identification of *S*. *pneumoniae* proteins

**Fig 4:**
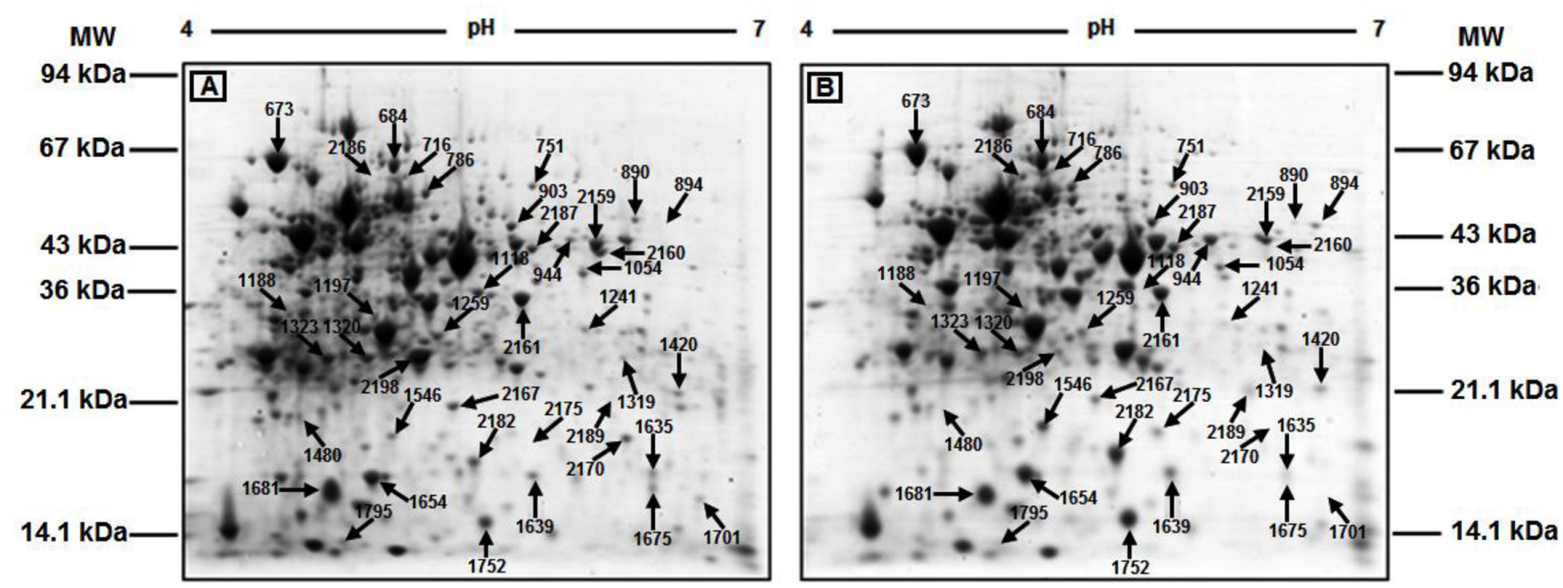
Annotated 2-DE protein profiles of an invasive site and non-invasive site pneumococcal isolate. Displayed are representative 2-DE protein profiles of the pneumococcal total cellular proteins extracted from an **(A)** invasive site isolate (B03) and a **(B)** non-invasive site isolate (E35) showing the pH range and the location of the molecular weight marker proteins. Protein spots, identified by LC-MS, are indicated using the Progenesis SameSpots^™^ assigned spot numbers.

**Fig 5:**
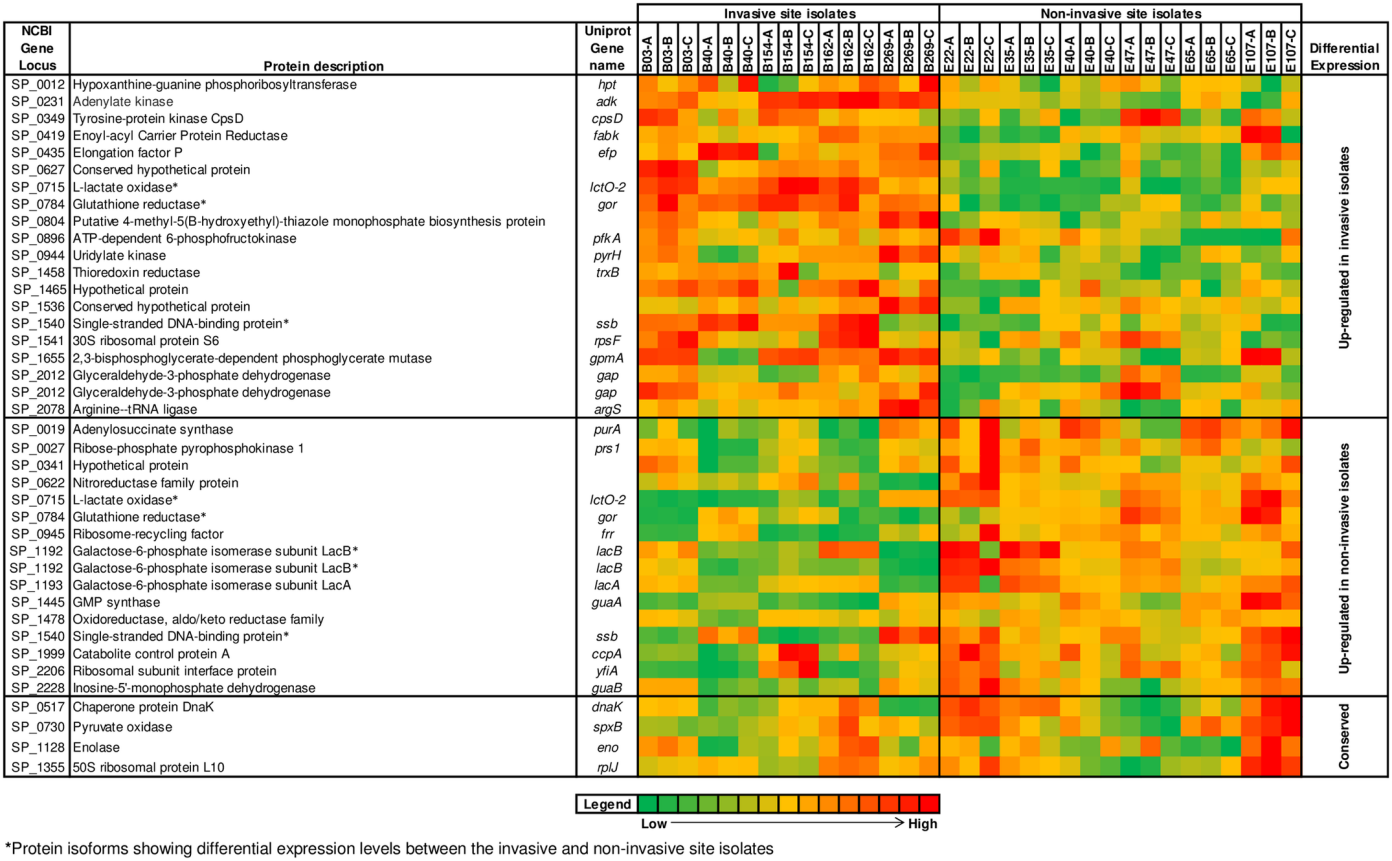
A heat map representation of the 40 protein spots identified by LC-MS. The identified protein spots showing conserved and differential abundance levels between the invasive and non-invasive site isolates were plotted on the heat map using the normalised spot volumes of the replicate gels of each isolate in the invasive and non-invasive group. The gene locus numbers are based on the publicly available *S*. *pneumoniae* TIGR4 genome annotation.

Forty selected protein spots that were either constant or variable in abundance between the invasive and non-invasive site isolates were successfully identified by liquid chromatography-mass spectrometry (LC-MS) against known *S*. *pneumoniae* proteins, based on significant MASCOT scores. The identified protein spots represented 32 unique pneumococcal proteins (Figs 3 and 4). Thirty-six of the identified protein spots were significantly different (ANOVA, p<0.05) in abundance between isolates for the two sites whereas 4 proteins (DnaK, SpxB, RplJ and Eno) showed no significant differences in abundance. Fold differences between the highest and lowest mean normalised volumes for the differentially expressed protein spots identified using the invasive group as a reference ranged between 0.25 to 4.0 (Fig 3). The normalised volumes of the protein spots for the identified proteins across the full series of 2-D gels are presented in the form of a heat map diagram in Fig 5. Twenty of the differentially expressed proteins identified were predominantly abundant in the invasive site isolates and 16 proteins showed the highest abundance in isolates from the non-invasive sites (Fig 3).

The migration of proteins in 2-DE gels can be altered by protein post-translational modifications (such as phosphorylation, glycosylation, acetylation and proteolysis) as well as degradation products and covalent reaction intermediates. These processes in addition to alternative splicing of mRNA and single-point mutations could also result in the generation of protein isoforms, in which protein products of the same gene have different observed *M*_*r*_ and *pI*. Four of the differentially expressed identified proteins were each present as two protein spots exhibiting different migrations consistent with them being isoforms of the same protein, which included Gor (Spots 890 and 894), LacB (spot 1639, 2182), LctO-2 (spots 944, 2159) and Ssb (spots 2170 and 2175) (Fig 3). Two protein spots (spots 1635 and 1675) with increased abundances in the invasive group were identified as Gap. However, the proteins observed *Mr* and *pI* values were significantly different from the corresponding values for Gap (Fig 3). Inspection of the identified peptides for Gap (S1 Table) indicates that they are derived from the N-terminus of the protein. This would be consistent with the protein spots identified as Gap being proteolytic degradation products of the intact protein. However, this was not the case for the protein spot identified as Eno, since the identified peptides were distributed throughout the intact Eno protein sequence (Fig 3, S1 Table). Consequently, the reason for the migration of the protein at an observed *Mr* of 15 kDa rather than the predicted 47 kDa is unknown.

The proteins were assigned to a number of functional groups, which included capsule biogenesis, DNA repair, protein deglycation, translation, stress response, virulence as well as amino acid, carbohydrate, lipid, and nucleotide metabolism. Those proteins with unknown functions including both hypothetical and conserved hypothetical proteins represented 13% of the identified differentially expressed proteins. Proteins predicted by PSORTb to be localised in the pneumococcal cell envelope (i.e. membrane and extracellular proteins) represented 8% of the identified differentially expressed proteins and all had significantly (ANOVA, p<0.05) higher abundances in the invasive site isolates compared to the non-invasive site isolates (Fig 3).

## Discussion

Proteomics is used extensively to characterize and compare the proteomes of pathogenic bacteria in order to understand infection mechanisms. In the present study, the comparison of clinical pneumococcal isolates by 2-DE protein expression profiling, has identified proteins associated with pneumococcal strain variability and site of infection. The selection of the bacterial isolates for analysis was primarily based on the bacterial sites of isolation, however, the serotypes (serotypes 4, 6, 9, 14 and 23) of the selected isolates are also clinically significant apropos of their disease potential, widespread geographical prevalence and antibiotic resistance [19,20]. The growth characteristics of the bacteria in BHI-FCS broth was studied to ensure standard *in vitro* growth conditions were used to avoid previously documented growth effects on proteome [13,21] and to ensure sufficient material were obtained for the proteomic analysis.

This report demonstrates that clinical *S*. *pneumoniae* isolates recovered from different sites within the host show characteristic cellular proteomes. Similar observations have been reported from proteomic analyses of *E*. *coli* and *H*. *pylori* [15–17]. Proteomic discrimination of invasive and non-invasive site isolates was due to the differential expression of 186 protein spots: 101 invasive site and 85 non-invasive site protein spots. Eighty one of these protein spots that were unique in discriminating the isolates by site of isolation provide an opportunity for determining the key activities of the bacteria that define the bacterial adaptation in blood and non-invasive sites of infection. Moreover, the clustering of the 2-D protein profiles revealed a greater heterogeneity among isolates in the invasive group compared to their non-invasive counterparts. This variability could be attributed to the differential adaptation of the bacteria to the challenging environment of the blood probably due to differences in the bacterial serotypes. Growing evidence suggests that pneumococci with different serotypes differ in their invasiveness [22,23]. Thus, the proteomic data presented in the current study could serve as a basis for defining the proteins that contribute to this variability.

Analysis of the bacterial protein profiles by PCA, according to the bacterial serotypes revealed the relatedness of the bacterial isolates as well as those proteins that displayed a serotype-specific pattern of expression. These proteomic data indicated a clustering of the isolates belonging to serotypes 9 and 14. This observation agrees with previous studies that used multilocus sequence typing to demonstrate that some pneumococcal serotype 14 isolates are variants of the 9V clone, especially on the basis of their antibiotic resistance profiles [24–26]. This suggests that the discrimination of the pneumococcal isolates by PCA may also reflect other phenotypic features of the bacteria such as antibiotic resistance.

Moreover, a higher number of proteins differed among the serotypes analysed compared to the isolation sites, indicating the high degree of diversity that exist among pneumococcal isolates [27,28]. Out of the 186 differentially expressed protein spots that discriminated the invasive and non-invasive site isolates, 105 proteins also differed among the pneumococcal isolates on the basis of their serotypes. These strain variable proteins can provide useful insights into defining the pathogenic variation that exists among clinical *S*. *pneumoniae* isolates.

Forty protein spots corresponding to 32 unique proteins were identified by LC-MS and functionally classified using the KEGG database. Metabolic proteins accounted for 50% of the differentially expressed proteins identified and the majority of these were involved in carbohydrate and nucleotide metabolisms. The ability of bacteria to respond to the diverse environments within the host is essential for their survival and metabolic proteins, particularly those involved in stress response and carbohydrate utilization, are central to pathogen infectivity [29–32].

One of the metabolic proteins identified as being upregulated in the invasive isolates was thioredoxin reductase (spot 1188). This enzyme is an ubiquitous antioxidant that plays an important role in the bacterial defence against extracellular oxidative stress and protects proteins from degradation or inactivation by reactive oxygen species (ROS) [33]. The enzyme acts by directly reducing oxidised cysteine or providing reducing equivalents to methionine sulfoxide reductase (Msr) enzymes, which reduce oxidized methionines [34,35]. Thioredoxin reductase enzymes have been extensively studied in *E*. *coli* where they play an anti-inflammatory role by suppressing neutrophil chemotaxis [36]. In the present study, there was a 1.6-fold increase in thioredoxin reductase abundance in the invasive compared to the non-invasive site isolates and the increased levels of ROS produced by host defences, such as the lactoperoxidase system, may account for the high levels of this protein in invasive site pneumococci.

The carbohydrate metabolic proteins, LacA (spot 1752) and LacB (spots 1639, 2182) showed 1.4- to 2.4-fold increase in abundance in the non-invasive site isolates. Glucose levels in the blood are generally higher than at other host sites infected by *S*. *pneumoniae* [31]. To thrive in low glucose host niches, pneumococci have developed metabolic mechanisms for pursuing alternative energy sources. Both LacA and LacB are involved in the tagatose-6-phosphate pathway, which is important for the utilization of galactose and lactose [37]. The expression of both enzymes is regulated by a global protein regulator, catabolite control protein A (CcpA; spot 2187), through a regulatory process called carbon catabolite repression. CcpA, which represents an important virulence determinant and metabolic regulator of sugar in *S*. *pneumoniae* [38,39] was upregulated in the non-invasive site isolates in this study. This protein was also detected with a *M*_*r*_ and *pI* that differed significantly from the predicted values for *M*_*r*_ and *pI*, suggesting it might undergo post-translational modification. Iyer and colleagues implicated CcpA in pneumococcal fitness during mucosal tissue infection by showing that *ccpA* mutant bacteria were attenuated in nasopharyngeal colonisation and infection of the lungs in murine models [39].

The capsular polysaccharide (CPS) biosynthesis protein, tyrosine-protein kinase (CpsD), was increased in abundance in the invasive site isolates. The regulation of CPS production through phosphorylation of the tyrosine residues in CpsD is crucial for the virulence of *S*. *pneumoniae* in mice [40,41]. *S*. *pneumoniae* undergoes spontaneous phase variation in CPS expression between an opaque and transparent form. The opaque phase is associated with increased capsule production and predominates in the blood [42]. The CpsD protein identified in the present study has a *pI* of 6.49 which differed from the theoretical *pI* of 8.96, thus suggesting a modification of the protein potentially by phosphorylation [43]. Tyrosine phosphorylated CpsD acts in concert with other CPS proteins (CpsB and CpsC) to regulate the assembly, transport and attachment of CPS to the pneumococcal cell wall [40,41,44,45]. According to Morona *et al*., [44,45] tyrosine phosphorylation of CpsD negatively regulates CPS production. In addition CpsD-deficient *S*. *pneumoniae* D39 bacteria produced a significant amount of CPS, but were unable to cause bacteremia in a murine model. However, Bender and colleagues [40] associated high levels of phosphorylated CpsD with increased CPS production in the *S*. *pneumoniae* D39 strain. Despite these contrasting findings on the precise role of tyrosine phosphorylation in the regulation of CPS production in *S*. *pneumoniae* D39 strain, the increased abundance of CpsD in the invasive site isolates in our study suggests a potential role in increasing CPS production. This proposal is consistent with the findings of the many studies that reported an association of increased CPS production with *S*. *pneumoniae* isolated from invasive human infections such as bacteraemia, sepsis and meningitis [42,46–48].

Among the proteins that showed no differences in abundance between the pneumococcal isolates were several proteins with well-documented roles in pneumococcal virulence and pathogenesis. These included the stress response proteins, DnaK and SpxB and the “moonlighting protein”, Eno. Both Eno and SpxB can induce protective immune responses in mouse infection models and could serve as potential immunogens for inclusion in a pneumococcal protein-based vaccine [49]. Although the pneumococcal DnaK protein has been considered as a vaccine candidate [50] it induces only a weak immune response in mice [49]. The low antigenicity of DnaK was due to the lack of surface-exposed epitopes on live intact pneumococcal cells [51]. PSORTb analysis of the inferred protein amino acid sequence derived from the UniProt database indicated DnaK to be of cytoplasmic origin.

The data presented in this study have demonstrated the potential of proteomics to not only discriminate clinical *S*. *pneumoniae* isolates recovered from invasive and non-invasive sites in the host, but also to identify candidate proteins that may be implicated in these two different aspects of the biology of *S*. *pneumoniae*. However, establishing the relationship between differential patterns of bacterial protein synthesis and the clinical phenotypes requires the inclusion of more pneumococcal isolates representing other serotypes and sites of isolation such as CSF, although isolates from these different niches may well have their own specific protein requirements. The proteomic analyses used in this study were biased towards the cytoplasmic proteins as evident by the small number of extracellular proteins identified. Given the significance of extracellular proteins in pneumococcal virulence and as potential vaccine antigens, their selective recovery and analysis will provide improved proteomic coverage to define further the clinical phenotypes. Further characterisation and confirmation of the proteins’ variation will be required using complementary techniques (for example by Western blotting or qPCR), although these approaches have limitations when investigating variable clinically relevant bacterial isolates as discussed elsewhere [15]. These difficulties will be increased in studies of genetically diverse bacteria such as *S*. *pneumoniae*. Nevertheless, the data presented above demonstrate the capacity of proteomics to differentiate clinical *S*. *pneumoniae* isolates according to their sites of isolation and to identify some of the proteins involved, and will lead to a better understanding of this significant human pathogen as well as the identification of new diagnostic and therapeutic markers.

## Materials and methods

### Origin of *S*. *pneumoniae* isolates

The *S*. *pneumoniae* isolates used in this study were selected from a collection of clinical pneumococcal isolates available from Aberdeen Royal Infirmary, Scotland; information was available on the isolate serotypes and sites of isolation [52]. The *S*. *pneumoniae* isolates characterised in the following study were isolated from “invasive” (blood) and “non-invasive” (sputum, tracheal aspirate, ear, eye and skin swabs) sites and included 5 different capsular serotypes of pneumococcus (Table 1). The bacterial isolates were originally serotyped by the Scottish Pneumococcal Reference Laboratory, Stobhill Hospital, Glasgow, United Kingdom, by coagglutination [53]. All of the *S*. *pneumoniae* isolates were stored at -70°C in microorganism preservation vials, PROTECT^™^ (Technical Service Consultants Ltd, Lancashire, UK) and recovered as described in the following section. The bacteria were grown on a blood agar plate together with a 5 μg Optochin disk to confirm their identity as *S*. *pneumoniae* by optochin sensitivity. The serotypes of the recovered isolates were confirmed by molecular serotyping as described in the Materials an methods section.

### Growth of *S*. *pneumoniae* isolates

Viable bacteria were recovered from the storage medium by inoculation onto 5% Columbia Blood Agar plates (E & O Laboratories Ltd, Bonnybridge, Scotland), and incubated at 37°C for 2–3 days in an anaerobic jar containing a CampyGen^™^ gas pack (Oxoid Ltd, Basingstoke, UK) for the generation of a microaerophilic atmosphere (5% Oxygen, 10% Carbon dioxide, and 85% Nitrogen). Between 20 and 30 *S*. *pneumoniae* colonies were passaged initially into 10 mL Brain Heart Infusion (BHI) broth (Oxoid, UK) supplemented with 5% (v/v) Foetal Calf Serum (FCS). The inoculated BHI-FCS broth medium was incubated stationary at 37°C for 15 h in an anaerobic jar containing a CampyGen^™^ gas pack. In order to generate a sufficient number of bacterial cells for the analysis of *S*. *pneumoniae* cellular proteins, a subculture in BHI-FCS broth from the first bacterial passage was performed. Briefly, 3 mL of bacterial suspension from the 10 mL broth culture (OD650 of 1.2) was added into 20 mL fresh BHI-FCS broth to give a starting OD650 of 0.1 and incubated stationary at 37°C under a microaerophilic atmosphere until an OD650 of 1.0 was reached.

### Monitoring the growth of *S*. *pneumoniae* isolates *in vitro*

The bacterial growth and viability were determined as follows. The *S*. *pneumoniae* isolates were grown in 20 mL BHI-FCS broth at 37°C in two independent biological replicates as previously described. Optical density measurements at 650 nm wavelength (OD650) were taken at 2 h intervals between 0 h and 16 h post-inoculation to determine the bacterial cell density. Serial 10-fold dilutions of the growth media ranging from 10^−1^ to 10^−7^ were prepared at times post-inoculation shown in Fig 1B to determine the bacterial viability. Five microliters of each dilution were plated onto blood agar plates and colony counts determined after 15 h incubation of the agar plates at 37°C in an anaerobic jar containing a CampyGen^™^ gas pack.

### Molecular capsular typing

The serotypes of the pneumococcal isolates used in this study were confirmed by PCR using primers published by the Centre for Disease Control (http://www.cdc.gov/streplab/pcr.html) shown in S2 Table. Bacterial DNA was extracted from one blood agar plate showing a confluent growth of bacteria by a boiling method. Briefly the bacterial cells were resuspended in 1 mL of molecular grade water and washed twice by centrifugation at 14000 x g for 10 min. The cell pellet was resuspended in 200 uL of molecular grade water and incubated in a boiling water bath for 10 min. The sample was cooled in ice for 5 min and then clarified at 14000 x g for 10 min. The extracted bacterial DNA was used in a duplex PCR reaction for the simultaneous detection of the serotype-specific genes and the pneumococcal capsular *wxg* gene, which was used as a positive control. Each 25 uL PCR reaction contained 12.5 uL of 2X MyTaq^™^ Red Mix (Bioline, UK), 500 nM of each primer and 25 ng bacterial DNA and was amplified using a GeneAmp^™^ PCR System 9700 thermal cycler (Applied Biosystems, USA) according to the following thermal cycling steps: 95°C for 5 min; 35 amplification cycles of 94°C for 30 s, 54°C for 1 min, and 72°C for 1 min; and a final extension step at 72°C for 7 min. The PCR products were separated on a 3% (w/v) Agarose LE gel (Sigma-Aldrich, UK) containing 10 uL of SYBR^®^ Safe DNA gel stain (Thermo Fisher Scientific, UK) at 110 volts for 45 min and visualised using a UV illuminator.

### Extraction of total bacterial cellular proteins and analysis by 2-DE

Whole cell protein extracts were prepared from three independent cultures of each pneumococcal isolate prepared as previously described. The bacterial cells were pelleted from the culture media by centrifugation at 2500 x g for 10 min and washed twice with sterile PBS. The cell pellets were resuspended in lysis buffer (7 M urea, 2 M thiourea, 4% (w/v) CHAPS, 0.3% (w/v) DTT, 1% (v/v) IPG buffer, pH gradient 4–7) (GE Healthcare, Buckinghamshire, UK) and incubated with lysozyme (10 μg/μL) and a nuclease cocktail containing DNase I (3 μg/μL) and RNase A (1.5 μg/μL) (Sigma-Aldrich, St. Louis, USA) for 30 min on ice. The enzyme treated cell suspensions were then frozen overnight at -20°C to enhance cell disruption. The treated cell suspensions were sonicated six times by continuous pulse at 90% power for 30 s each using an Ultrason 250 Sonicator (LabPlant, UK) fitted with a microprobe. To minimise protein degradation by proteolysis, the cell lysates were cooled for 30 s on ice between each pulse of sonication. Finally, the cell lysates were clarified at 16200 x g for 10 min and the soluble proteins in the supernatants concentrated using the ReadyPrep^™^ 2-D Cleanup Kit (Bio-Rad Laboratories Ltd, Hertfordshire, UK) according to the manufacturer’s instructions. The protein concentrations were determined by RC DC Protein assay using BSA (Bio-Rad Laboratories, Hercules, USA) as the standard. The bacterial proteins (100 μg) were analysed by 2-DE using small format 2-DE gels according to the protocol described by Cash and Argo [54]. The proteins were separated on 7 cm IPG gel strips (Bio-Rad Laboratories Ltd, Hertfordshire, UK) with a pH gradient of 4–7 in the first dimension and the second dimension separation was carried out on 12% single concentration polyacrylamide slab gels (Bio-Rad Laboratories Ltd, Hertfordshire, UK). The resolved proteins were detected by Colloidal Coomassie Blue G250 staining as described by Cash and Argo [54].

### Imaging and analysis of the 2-DE protein profiles

The 2-DE protein profiles were scanned at 600 dpi using an Image Scanner III (GE Healthcare Ltd, UK) supported by Labscan software. The 16 bit gel image files were transferred to the Progenesis SameSpots^™^ software, version 4.5 (Nonlinear Dynamics, Newcastle, UK) for spot detection and analysis as a single data set according to the method described by Smith *et al*. [15]. Briefly, gel images representing three biological replicates for each bacterial isolate were reviewed and cropped as required. A reference gel image was selected for the data set of 2-DE gel images. The remaining gels were sequentially matched against the reference gel and spots on the 2-DE profiles were detected and transformed into normalised abundances using the inbuilt routines provided by the Progenesis SameSpots^™^ software. The detected spots were automatically assigned numbers by the software and these are used to refer to the spots in the text. Statistical differences in normalised spot abundance between the groups were detected by ANOVA (p<0.05) using the software’s statistical package. Further analysis of the data by Principal Component Analysis (PCA), using the inbuilt software routines was carried out as described by Smith *et al*. [15]. Fold-differences in the mean normalised protein spot volumes between the invasive and non-invasive site isolates were calculated relative to the values for the invasive group, which was used as a reference.

### In-Gel protein digestion

Protein spots, selected according to the criteria described in the Results section, were excised as 1 mm diameter gel plugs from dried Coomassie Blue stained 2-DE gels and transferred into a 96 well reaction plate for in-gel digestion using a ProGest digestor robot (Genome solutions, Huntingdon, UK). The gel plugs were first equilibrated in water, destained with acetonitrile and then sequentially washed in 25mM ammonium bicarbonate (AMBIC), acetonitrile and 50 mM AMBIC. The gel pieces were finally dehydrated with acetonitrile and the cysteine residues of the proteins reductively alkylated by incubating for 10 mins at 60°C in 30 μL reducing solution (10 mM dithiothreitol in 50 mM AMBIC), followed by incubation at room temperature (RT) for 15 min in 30 μL alkylating solution (55 mM iodoacetamide in 50 mM AMBIC). This was followed by washing and drying of the gel pieces with 50 mM AMBIC and acetonitrile respectively. The proteins within the gel pieces were digested with 15 μL trypsin (20 ng/μl in sequencing grade water, Promega, UK) for 8 h at 37°C. The peptide digests were eluted in a mixture of 10% formic acid and acetonitrile and dried in a SpeedVac (Savant, USA). The dried peptides were reconstituted in 50 μL loading buffer (98% water, 2% acetonitrile and 0.1% formic acid) and dissolved at RT by shaking for 15 mins at 1800 rpm. The tryptic peptide digests were clarified at 18,800 x g for 5 min and the peptides analysed by LC-MS.

### Protein identification by LC-MS

The tryptic peptide digests were analysed using an UltiMate 3000 ultra-high-pressure nano HPLC system (Dionex (UK) Ltd., Camberley, Surrey, UK) interfaced with a HCTultra PTM Discovery System (Bruker Daltonics Ltd., Coventry, UK). The peptides were separated on a PepSwift monolithic PS-DVB capillary column (200 μm i.d. x 5 cm; Dionex (UK) Ltd.) at a flow rate of 2.0 μL/min using a linear gradient of acetonitrile provided by solvent A (3% acetonitrile in 0.05% formic acid) and solvent B (80% acetonitrile in 0.04% formic acid). The peptide separation was carried out using a gradient of 3–45% solvent B for 12 minutes, followed by a column wash in 90% solvent B for 1 minute and then equilibration in 3% solvent B for 5 minutes. MS/MS data, averaged from two spectra, with a scan range of 100–2200 m/z, were acquired in data-dependent Auto MS2 mode. Up to 3 precursor ions were selected from the MS scan (range 300–1500 m/z, averages 3) in each Auto MS2 cycle. Precursors were actively excluded after selection twice within a 1.0 min window, and singly-charged ions were also excluded. Peptide peaks were detected and deconvoluted automatically using Data Analysis Software (Bruker). Mass lists in the form of Mascot Generic Files were created automatically and used as the input for the MS/MS ion searches in the NCBIprot database. The search was performed by a local MASCOT server (version 2.2) using the Matrix Science web server (www.matrixscience.com) and the search parameters were as follows: Taxonomy—S. pneumoniae; Enzyme—Trypsin; Fixed modifications—Carbamidomethyl (C); Variable modification—Oxidation (M); Mass values—Monoisotopic; Peptide mass tolerance– 1.5 Da; Fragment mass tolerance– 0.5 Da; Max missed cleavages– 1; Peptide charge– 2+ and 3+; Instrument type—ESI-TRAP. The MASCOT significance threshold p<0.05 was used for peptide identification and a false discovery rate (FDR) of less than 1% using a target decoy search was used for the peptide validation.

### Bioinformatic analysis of identified proteins

The identified proteins were categorised into their respective biological functions using the Kyoto Encyclopedia of Genes and Genome (KEGG) database (http://www.genome.jp/kegg/pathway.html). The association of the proteins with the KEGG pathways was determined using *S*. *pneumoniae* TIGR4 as the reference organism. The subcellular localizations of the identified proteins were predicted using the web-based algorithm PSORTb 3.0 (http://www.psort.org/psortb/index.html). Since PSORTb cannot predict lipoprotein motifs, the subcellular localizations of the proteins predicted as unknown by PSORTb were further characterised using CELLO2GO (http://cello.life.nctu.edu.tw/cello2go/).

## Supporting information

S1 Table Peptides identified by LC-MS analysis of selected *S*. *pneumoniae* proteins. (PDF)

S2 Table Primers used for the molecular capsular typing. (TIF)

S1 Fig Representative global proteome profiles of invasive (B) and non-invasive (E) site isolates grown in BHI-FCS broth and resolved on small format 2-D gels. (TIF)

## Abbreviations

2-DE: two-dimensional gel electrophoresis
LC-MS: liquid chromatography-mass spectrometry
PCA: Principal Component Analysis
