# Supplementary material for: Proteomic variation and diversity in clinical Streptococcus pneumoniae isolates from invasive and non-invasive sites
Source: PLoS One. 2017 Jun 2;12(6):e0179075. doi: 10.1371/journal.pone.0179075 (PMC5456405; doi:10.1371/journal.pone.0179075)
Supplement: S1 Table — (PDF) [file pone.0179075.s001.pdf]

Spot #	Identified NCBI Accession #	Protein description	AA Residues	Start AA	End AA	Matched Peptide sequence	Unique peptide (U)
673	WP_055327301.1	Chaperone protein DnaK	607	2	25	SKIIGIDLGTTNSAVAVLEGTESK	
				4	25	IIGIDLGTTNSAVAVLEGTESK	
				26	34	IIANPEGNR	
				44	54	NGEIIVGDAAK	
				83	98	EYTPQEISAMILQYLK	
				99	108	GYAEDYLGEK	
				112	125	AVITVPAYFNDAQR	
				134	141	IAGLEVER	
				142	157	IVNEPTAAALAYGLDK	
				197	205	LGGDDFDQK	
				206	216	IIDHLVAEFKK	
				316	324	IPAVVEAVK	
				427	437	FQLTDIPAAPR	
				438	450	GIPQIEVTFDIDK	
				438	457	GIPQIEVTFDIDKNGIVSVK	
				507	518	NEVDQAIFATEK	
				533	544	DAAQAALDDLKK	
684	WP_000191791.1	Pyruvate Oxidase	591	6	17	ITASAAMLNVLK	U
				52	65	HEETGALAAVMQAK	U
				106	132	LVNELNMDAFQELNQNPMYNGIAVYNK	U
				134	142	VAYAEQLPK	U
				185	198	SFIAPALNEVEIDK	U
				220	229	AGEVITELSR	U
				296	305	FIQVDIDPYK	U
				310	325	HALDASILGDAGQAAK	U
				361	376	TEGELQLYQVYNAINK	U
				377	397	HADQDAIYSIDVGNTTQTSTR	U
				409	427	TSPLFATMGIALPGGIAAK	U
				484	497	HLFGVDFTNADYAK	U
				498	511	IAEAQGAVGFTVDR	U
				512	524	IEDIDAVVAEAVK	U
				566	577	EKYEAEELVPFR	U
				568	577	YEAEELVPFR	U
				578	588	LFLEEEGLQSR	U
716	ANO37856.1	Arginine-tRNA ligase	563	32	47	NSEMGDIAFPAFSLAK	
				51	63	KAPQMIAAELAEK	
				52	63	APQMIAAELAEK	
				72	85	VVATGPYVNFFLDK	
				86	100	SAISAQVLQAVTTEK	
				164	173	QFGMLIVAYK	
				233	241	DESLVEFNR	
				248	262	VEFDSYNGEAFYNDK	
				263	273	MDAVVDILSEK	
				274	289	GLLLESEGAQVVNLEK	U
				301	310	SDGATLYITR	
				327	340	SIYVVGQEQSAHFK	
				377	392	GNVILLEPTVAEAVSR	
				498	509	AADNFEPSIIAK	
				510	520	FAISLAQSFNK	
				538	549	LALSYATAVVLK	
				554	563	LLGVEAPEKM	
751	WP_000743621.1	Hypothetical protein	494	4	16	QAFSSEQYLNLQR	
				23	37	INQFDGKLYLEFGGK	
				65	82	EQVEVVIAINASNIEHSK	
				85	97	GDLGISYDQEVLR	

				102	127	FNELGIFVGSVVITQYAGQPAADAFR	
				170	183	NLIVVTAPGPGSGK	
				308	316	YYQTVLDFK	
				327	342	KIELLMNDLGITPADR	
				328	342	IELLMNDLGITPADR	
				374	390	NSELFGPTAAALINAIK	
				425	446	LHSNEILIALAITATENPDAAR	
				456	474	GSEAHSTIILTDEDKNVLR	
				475	490	KLGINVTFDPYYQYDR	
				476	490	LGINVTFDPYYQYDR	
786	CIW04908.1	Inosine-5'-monophosphate dehydrogenase	484	114	126	ISGVPVVETLENR	
				180	196	LPLVDEEGSLSGLITIK	
				180	200	LPLVDEEGSLSGLITIKDIEK	
				201	214	VIEFPNAAKDEFGR	
				215	230	LLVAGAVGVTSDTFER	
				269	282	TLIAGNIATAEGAR	
				283	293	ALYEAGVDVVK	
				294	305	VGIGPGSICTTR	
				306	326	VIAGVGVPQVTAIYDAAAVAR	
				399	409	YFQGSVNEANK	
				423	437	GAAADIVFQMIGGIR	
				450	466	ELHDNAQFIEMSGAGLK	U
890	COG61030.1	Gluthatione Reductase	448	3	21	EYDIIAIGGGSGGIATMNR	U
				37	49	LGGTCVNVGCVPK	
				73	84	TTDLNFDFATLR	U
				204	215	GFDSYIVEGLVK	
894	WP_050265245.1	Gluthatione Reductase	448	3	21	EYDIIAIGGGSGGIATMNR	
				36	49	KLGGTCVNVGCVPK	
				37	49	LGGTCVNVGCVPK	
				73	84	TTDLNFDFATLR	
				204	215	GFDSYIVEGLVK	
				323	331	LFNGKTTSK	
				371	382	SSFASMYSACTR	
				428	448	ADFDATVAIHPTSSEEFVTMR	
903	WP_001832534.1	Adenylosuccinate synthetase	428	2	16	TSVVVVGTQWGDEGK	
				19	32	ITDFLSANAEVIAR	
				33	48	YQGGDNAGHTIVIDGK	
				65	77	ISVIGNGMVVNPK	
				82	97	ELSYLHEEGVTTDNLR	
				102	114	AHVILPYHIELDR	
				147	157	IADLLDKDIFR	
				199	215	YVTDTSVILNDALDNGK	
				199	216	YVTDTSVILNDALDNGKR	
				272	288	VGDGPFPTELFDEVGER	
				306	315	VGWFDSVVMR	
				342	352	ICVAYDLDGQR	
				353	364	IDYYPASLEQLK	
				366	384	CKPIYEELPGWSEDITGVR	
				408	417	ISTFSVGPGR	
944	WP_050279272.1	Lactate oxidase	371	31	47	AAFGYIASGAEDTFTLR	
				94	105	LANEQGEVATAR	
				163	176	AIVLTADATVGGNR	
				220	235	DVEFIAEYSGLPVYVK	
				246	262	SLAAGASGIWVTNHGGR	
				263	283	QIDGGPAAFDSLQEVAEAVDR	U
				284	294	RVPIVFDSGVR	

				285	294	VPIVFDSGVR	
				341	355	TVMQLSGAQTIEDVK	
				361	371	HNPYNPTFPVD	U
1054	WP_000010156.1	Ribose-phosphate pyrophosphokinase 1	326	8	15	LFALSSNK	U
				21	31	VAQEIGIELGK	U
				36	51	QFSDGEIQVNIEESIR	U
				82	98	ASAESVNVVMPYYGYAR	U
				113	125	LVANMLEVAGVDR	
				160	179	RGMVGSDYVVVSPDHGGVTR	U
				161	179	GMVGSDYVVVSPDHGGVTR	U
				199	214	SVDKMNTSEVMNIIGK	U
				203	214	MNTSEVMNIIGK	U
				290	305	IEQISIAHLLGDAIVR	U
1118	EJH00351.1	Enoyl-acyl Carrier Protein Reductase	316	2	26	KIDYPIFQGGMAWVADGDLAGAVSK	U
				3	26	IDYPIFQGGMAWVADGDLAGAVSK	
				27	40	IDYPIFQGGMAWVADGDLAGAVSK	
				83	93	VVTTGAGNPSK	
				121	139	IGADAVIAEGMEAGGHIGK	
				231	341	DFELAEKDAFK	
				242	260	QEDPDLEIFEQMGAGALAK	
				261	283	AVVHGDVDGGSVMAGQIAGLVSK	
				284	300	EETAEEILKDLYYGAAK	
1188	EOB33722.1	Thioredoxin reductase	303	126	139	GVSYCAVCDGAFFR	U
				191	200	ISFIWDSVVR	U
				204	217	GENRVESVVFENVK	U
				208	217	VESVVFENVK	U
				280	297	QVTTAVGDGAIAGQEAYK	U
1197	WP_001099664.1	Oxidoreductase, aldo/keto reductase family	280	1	21	MNTYQLNNGVEIPVLGFGTFK	U
				42	60	HIDTAAIYQNEESVGQAIK	U
				171	185	LAPGVYQEEVVAYCR	U
				186	206	EKGILLEAWGPFGQGELFDSK	U
				188	206	GILLEAWGPFGQGELFDSK	U
				218	237	SVAQIALAWSLAEGFLPLPK	U
1241	WP_000390780.1	Conserved hypothetical protein	249	13	22	INQLFSTDIK	
				28	41	EVFSYSVDSVLLSR	
				69	81	TQAQILSVEIQER	
				118	129	VDMILCNPPYFK	
				179	188	LLDILDTLKR	
1259	WP_031222472.1	Uridylate kinase	245	13	22	LSGEALAGER	
				23	36	GVGIDIQTVQTIAK	
				37	59	EIEEVHSLGIEIALVIGGGNLWR	U
				101	116	VQTAIAMQQVAEPYVR	
				126	149	GRIVIFGAGIGSPYFSTDTTAALR	
				150	162	AAEIEADAILMAK	U
				163	174	NGVDGVYNADPK	
				227	244	VVFGENIGTTVSNNIEEK	
				227	245	VVFGENIGTTVSNNIEEKE	
1319	WP_050092849.1	Tyrosine-protein kinase CpsD	227	37	49	VISVTSVNPGEGK	U
				169	184	CDASILVTATGEANKR	U
				199	207	LFLGVVLNK	U
1320	WP_001050429.1	Adenylate kinase	212	1	13	MNLLIMGLPGAGK	U
				79	89	ETGFLLDGYPR	U

				168	183	LDVNIAQGEPIIAHYR	U
				186	206	GLVHDIEGNQDINDVFSDIEK	U
1323	ANO36350.1	Elongation factor P	186	9	19	AGMTFETADGK	U
				47	60	TGSTFDTSYRPEEK	U
				61	76	FEQAIIETVPAQYLYK	U
				173	185	LVINTAEGTYVSR	U
1420	CYK05467.1	Nitroreductase family protein	160	53	69	VGGANNFSEEQLQYFMK	
				118	129	SKVNEVLEIEDR	
				120	129	VNEVLEIEDR	
				130	144	FRPELLITVGYTDEK	U
				151	159	LPVDEIIEK	U
1480	EHE10146.1	Putative 4-methyl-5(B-hydroxyethyl)-thiazole monophosphate biosynthesis protein	167	10	32	ANITCDMVGFEEQVTGSHAIQVR	
				61	77	DNQTLIQELQSFEQEGK	U
				79	96	LAAICAAPIALNQAEILK	
				118	130	ETVVVDGQLTTSR	
1546	EOB34484.1	Ribosomal subunit interface protein	182	4	18	YSIRGENLEVTEAIR	
				8	18	GENLEVTEAIR	
				8	24	GENLEVTEAIRDYVVSK	
				31	41	YFQPEQELDAR	
				51	66	TAKVEVTIPLGSITLR	
				67	84	AEDVSQDMYGSIDLVTDK	U
				169	180	REDGEIGLLEVK	
				170	182	EDGEIGLLEVKES	
1635	CVY04434.1	Glyceraldehyde-3-phosphate dehydrogenase	326	5	12	VGINGFGR	U
				21	31	IQNVEGVEVTR	U
				54	61	FDGTVEVK	U
1639	COH06448.1	Galactose-6-phosphate isomerase subunit LacB	152	3	15	IAIGCDHIVTDEK	
				16	23	MAVSEFLK	
				24	39	SKGYEVIDFGTYDHTR	
				26	39	GYEVIDFGTYDHTR	
				48	77	KVGEAVTSGQADLGVCICGTGVGINNAVNK	U
				97	109	EQLNANVIGFGGK	
1654	WP_001287280.1	50S ribosomal protein L10	166	9	19	KAELVDVVAEK	
				22	32	AAASIVVVDAR	
				33	43	GLTVEQDTVLR	
				68	95	AGLEDLASVFVGPSAVAFSNEDVIAPAK	U
				103	110	NAEALEIK	
				111	121	GGAIEGAVASK	
				111	133	GGAIEGAVASKEEILALATLPNR	
				122	133	EEILALATLPNR	
				134	149	EGLLSMLLSVLQAPVR	U
				134	149	EGLLNMLLSVLQAPVR	U
1675	CVY04434.1	Glyceraldehyde-3-phosphate dehydrogenase	326	5	12	VGINGFGR	U
				21	31	IQNVEGVEVTR	U
				32	46	INDLTDPVMLAHLLK	U
				54	61	FDGTVEVK	U
				117	129	KVVITAPGGNDVK	U

1681	WP_000022813.1	Enolase	434	17	34	GNPTLEVEVYTESGAFGR	U
				106	120	LGANAILGVSIAVAR	
				313	330	VQLVGDDFFVTNTDYLAR	U
				331	343	GIQEGAANSILIK	U
				344	361	VNQIGTLTETFEAIEMAK	
				373	394	SGETEDSTIADIAVATNAGQIK	U
				413	424	IEDQLGEVAEYR	
1701	WP_000858727.1	Hypothetical protein	148	108	125	LYHQTLDLIDHTQEEVIQ	U
1752	COH06417.1	Galactose-6-phosphate isomerase subunit LacA	141	2	13	SIVIGADAAGLR	U
				75	87	IKGMVAAEVSDER	U
				77	87	GMVAAEVSDER	U
				100	114	MITMGAQLVGDELAK	U
1795	WP_050216039.1	30S ribosomal protein S6	96	26	41	FDSILTDNGATVVESK	
				48	56	LAYEIKDFR	U
				57	74	EGLYHIVNVEANDDAALK	
2159	WP_050229519.1	L-lactate oxidase	378	31	47	AAFGYIASGAEDTFTLR	
				80	93	LSSPIIMAPVAAHK	
				94	105	LANEQGEVATAR	
				163	176	AIVLTADATVGGNR	
				220	235	DVEFIAEYSGLPVYVK	
				246	262	SLAAGASGIWVTNHGGR	
				263	283	QIDGGPAAFDSLQEVAEAVDR	
				285	294	VPIVFDSGVR	
				341	355	TVMQLSGAQTIEDVK	
2160	WP_001077203.1	Conserved hypothetical protein	345	10	22	ELTAIASPTGFTR	
				31	41	TLEGFGYQPVR	
				45	52	GGVNVTIK	
				61	74	YVTAHVDTLGAIVR	
				154	168	ALGIEVGDFISFDPR	
				169	178	TVVTDTGFIK	U
				186	196	VSAAILLNLLR	
				285	306	LDIYPFYGSDASAAMSAGAEVK	
				307	322	HALLGAGIESSHSYER	U
				323	333	THIDSVVATER	
2161	EOB16580.1	ATP-dependent 6-phosphofructokinase	335	4	21	IAVLTSGGDAPGMNAAIR	
				73	84	YPEFAQLEGQLK	
				92	111	HGIEGVVVIGGDGSYHGAMR	
				162	171	RTFVIEVMGR	
				163	171	TFVIEVMGR	
				172	197	NAGDIALWAGIATGADEIIIPEAGFK	
				198	206	MEDIVASIK	
				214	233	KHNIIVLAEGVMSAAEFGQK	
				215	233	HNIIVLAEGVMSAAEFGQK	
				236	244	EAGDTSDLR	
				245	253	VTELGHIQR	
				278	288	EGIGGVAVGIR	
				292	314	MVENPILGTAEEGALFSLTAEGK	
				323	332	ADIELSSLNK	
2167	WP_000892184.1	Hypoxanthine-guanine phosphoribosyltransferase	180	9	21	VLVSHDEITEAAK	
				30	43	DYAGKNPILVGILK	
				35	43	NPILVGILK	

				125	135	IATLLDKPEGR	
				166	180	NLPYIGVLKEEVYSN	U
2170	WP_000609600.1	Single-stranded DNA-binding protein	156	1	10	MINNVVLVGR	
				2	10	INNVVLVGR	
				19	35	YTPSNVAVATFTLAVNR	
				45	55	EADFINVVMWR	
				67	76	KGSLIGVTGR	
				68	76	GSLIGVTGR	
				90	105	VYVTEVVAENFQMLER	U
				137	156	NENPFGATNPLDISDDDLPF	U
2175	WP_016397693.1	Single-stranded DNA-binding protein	156	1	10	MINNVVLVGR	
				19	35	YTPSNVAVATFTLAVNR	U
				45	55	EADFINVVMWR	U
				67	76	KGSLIGVTGR	U
				90	106	VYVTEVVAENFQMLESR	U
				137	156	DENPFGATNPLDISDDDLPF	U
2182	WP_061364440.1	Galactose-6-phosphate isomerase subunit LacB	171	3	15	IAIGCDHIVTDEK	U
				24	39	SKGYEVIDFGTYDHTR	U
				26	39	GYEVIDFGTYDHTR	U
				97	109	EQLNANVIGFGGK	U
				148	163	NAQQTDANFFTEFLEK	U
2186	WP_000065727.1	GMP synthase	520	14	29	IIVLDYGSQYNQLISR	U
				228	247	VLLGLSGGVDSSVVGVLLQK	
				308	323	IIGNEFVYVFDDEASK	
				329	351	FLAQGTLYTDVIESGTDTAQTIK	
				469	482	AITSIDGMTADFAK	
2187	WP_000990602.1	Catabolite control protein	336	1	14	MNADDTVTIYDVAR	
				15	25	EAGVSMATVSR	U
				41	47	VLEVIDR	
				85	94	GIDDIAEMYK	
				95	108	YNIVLANSDEDNEK	U
				109	119	EVSVVNTLFSK	U
				183	197	IAFVSGPLVDDINGK	
				209	223	KAGITYSEGLVFESK	U
				210	223	AGITYSEGLVFESK	U
				224	235	YSYDDGYALAER	
				263	282	GVSVPEDFEIITSDDSQISR	
				319	329	EVLLPHGLTER	
2189	COO10712.1	Ribosome-recycling factor	185	64	72	VLLVTPFDK	U
				100	110	LVIPALTEETR	U
2198	CJC06826.1	Phosphoglyceromutase	230	17	31	ANLFTGWADVDLSEK	
				32	41	GTQQAIDAGK	
				45	59	EAGIEFDQAYTSVLK	U
				64	81	TTNLALEASDQLWVPVEK	
				99	114	AEAAEQFGDEQVHIWR	
				138	153	YASLDDSVIPDAENLK	
				176	187	NVFVGAHGNSIR	
				220	230	LNVVSEYYLGK	
