# Supplementary figures and images for: Proteomic variation and diversity in clinical Streptococcus pneumoniae isolates from invasive and non-invasive sites
Source: PLoS One. 2017 Jun 2;12(6):e0179075. doi: 10.1371/journal.pone.0179075 (PMC5456405; doi:10.1371/journal.pone.0179075)

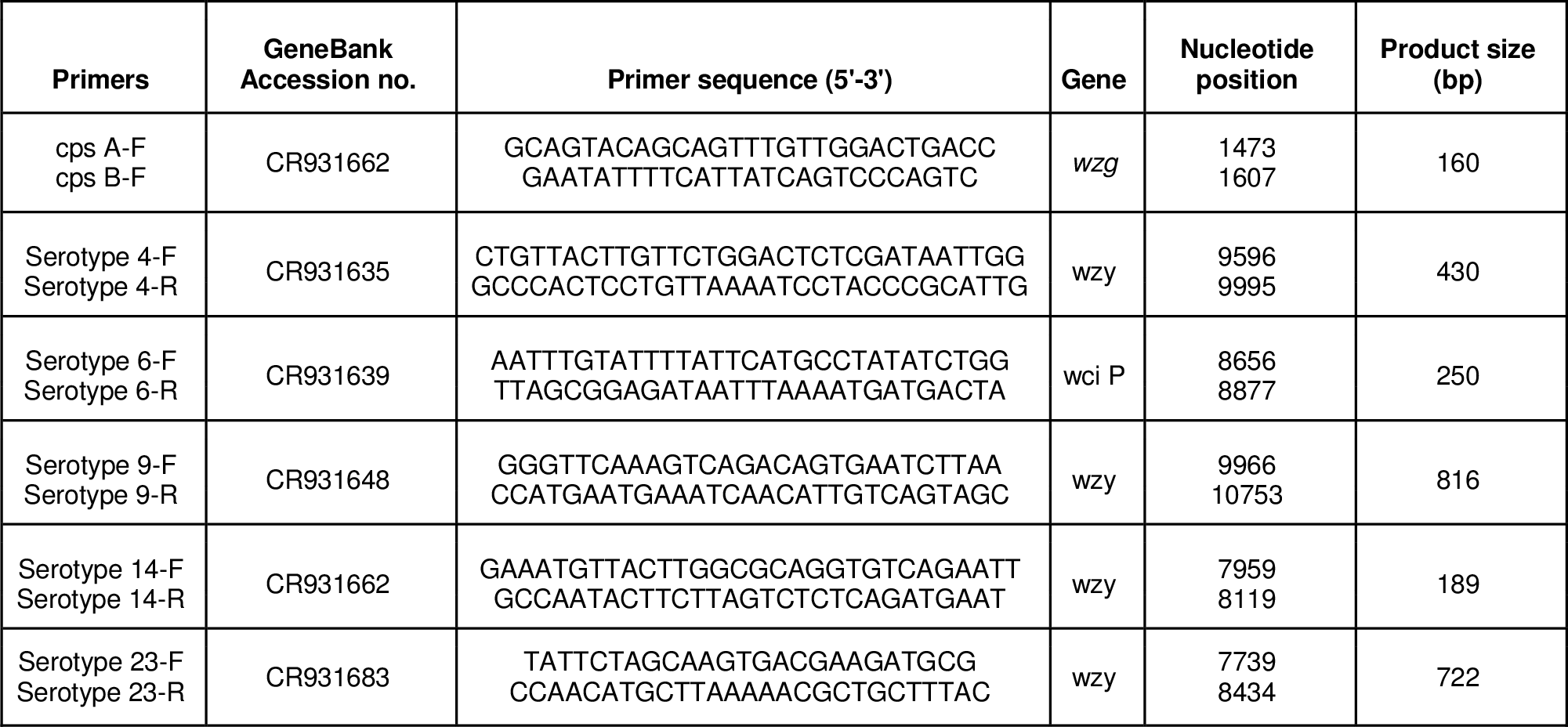

Supplement: S2 Table — (TIF) [file pone.0179075.s002.tif]

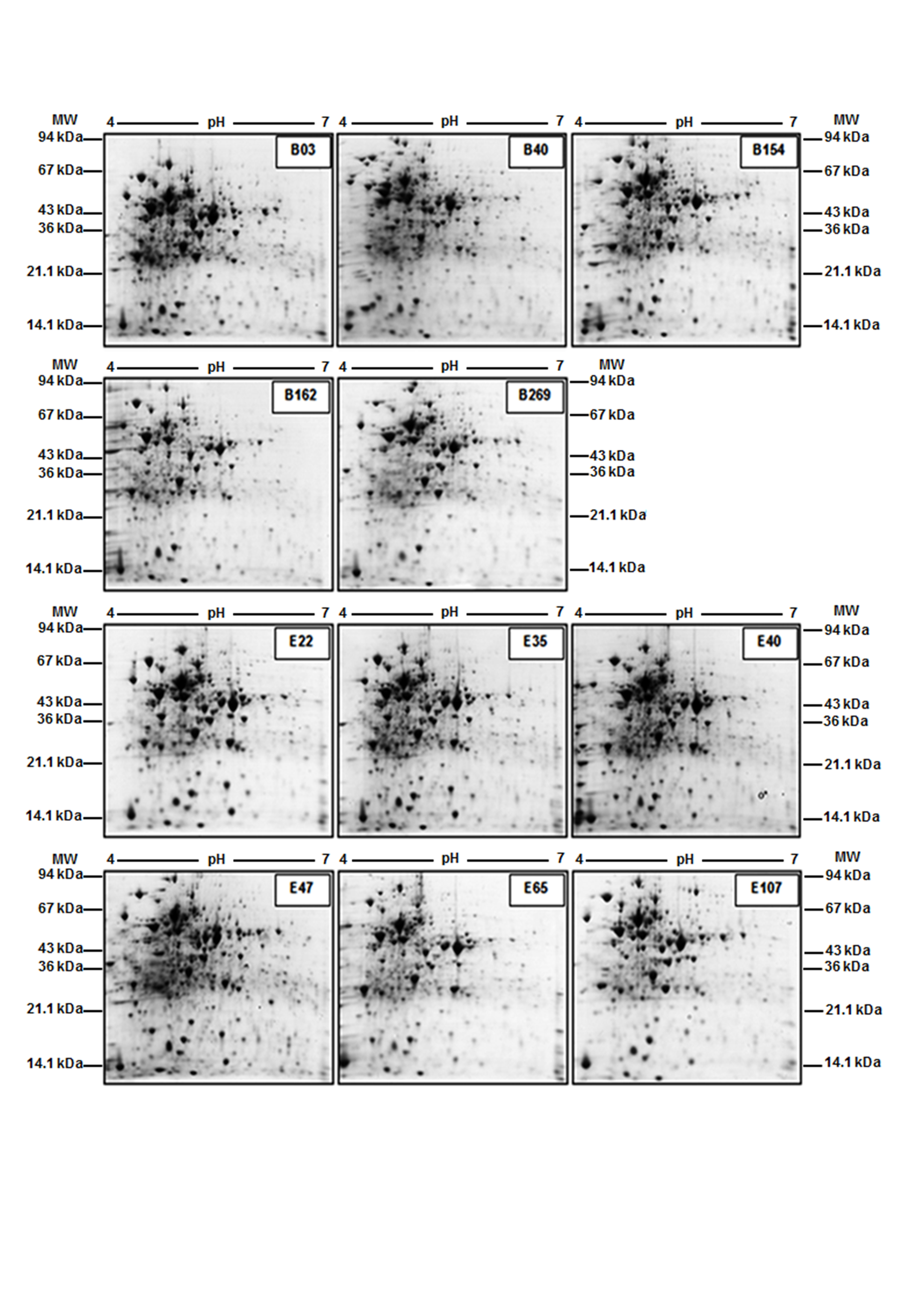

Supplement: S1 Fig — (TIF) [file pone.0179075.s003.tif]
